# An Experimental Approach for Optimizing Coating Parameters of Electroless Ni-P-Cu Coating Using Artificial Bee Colony Algorithm

**DOI:** 10.1155/2014/976869

**Published:** 2014-10-29

**Authors:** Supriyo Roy, Prasanta Sahoo

**Affiliations:** Department of Mechanical Engineering, Jadavpur University, Kolkata 700032, India

## Abstract

This paper aims to present an experimental investigation for optimum tribological behavior (wear depth and coefficient of friction) of electroless Ni-P-Cu coatings based on four process parameters using artificial bee colony algorithm. Experiments are carried out by utilizing the combination of three coating process parameters, namely, nickel sulphate, sodium hypophosphite, and copper sulphate, and the fourth parameter is postdeposition heat treatment temperature. The design of experiment is based on the Taguchi L_27_ experimental design. After coating, measurement of wear and coefficient of friction of each heat-treated sample is done using a multitribotester apparatus with block-on-roller arrangement. Both friction and wear are found to increase with increase of source of nickel concentration and decrease with increase of source of copper concentration. Artificial bee colony algorithm is successfully employed to optimize the multiresponse objective function for both wear depth and coefficient of friction. It is found that, within the operating range, a lower value of nickel concentration, medium value of hypophosphite concentration, higher value of copper concentration, and higher value of heat treatment temperature are suitable for having minimum wear and coefficient of friction. The surface morphology, phase transformation behavior, and composition of coatings are also studied with the help of scanning electron microscopy, X-ray diffraction analysis, and energy dispersed X-ray analysis, respectively.

## 1. Introduction

Electroless coatings have gained wide acceptance due to their excellent corrosion and wear resistance properties [1]. Electroless coating, also known as chemical or autocatalytic coating, is a nongalvanic plating method that involves several simultaneous chemical reactions in an aqueous solution, which occur without the use of external electrical power. This makes this process different from conventional electroplating process which requires external current source. In recent days, the binary electroless Ni-P coatings have become the research focus due to their superior tribological properties. These properties can be further improved by incorporating a third particle into the binary alloy. The choice of the third particle depends on the desired property. Ternary Ni-P coatings, such as Ni-Cu-P [2, 3], Ni-W-P [4–8], Ni-P-PTFE [9, 10], Ni-P-SiC [11, 12], Ni-P-Al_2_O_3_ [13, 14], and Ni-P-TiO_2_ [15–17], have been investigated by different researchers. Among the ternary Ni-P alloy coatings, the electroless Ni-Cu-P alloy presents superior corrosion resistance and thermal conductivity than others [18–23]. But, only good corrosion resistant property is not sufficient for industrial purposes. In practical situations, both corrosion and wear take place simultaneously, as in case of bearing of pumps and turbines. Thus, in order to protect these components, a coating is needed, which should have both anticorrosive and good wear-resistant properties. The electroless Ni-P-Cu coating has good anticorrosive property. Liu and Zhao [24] studied the corrosive property of electroless Ni-P-Cu to compare it with binary Ni-P coating at different corrosive atmosphere and it reveals that Ni-P-Cu coating shows better anticorrosive property than binary Ni-P coating in HCL and NaCl medium. A similar comparative study was performed by Wang et al. [25] among the electroless Ni-P-Cu coating, Ni-P coating, and stainless steel and it was found that at 50% NaOH solution medium the Ni-P-Cu coating shows better anticorrosive property than others.

The review of the existing literature reveals that friction and wear behavior of Ni-P-Cu coatings have not been studied so far and no data is available on optimization of the tribological behavior of this coating. Hence, the present study considers friction and wear behavior of this coating. Also an attempt is made to find out the optimum combination of coating process parameters of electroless Ni-P-Cu coating for minimum friction and wear. Friction and wear appear to be similar in their physical behavior since both are loss outputs from any tribological system. Friction is the resistance to motion, while wear refers to primarily material loss. However, friction and wear are not interrelated, rather two distinct system responses. Low friction does not necessarily mean low wear or vice versa. Thus, both friction and wear need to be minimized for better tribological performance. Several multiresponse optimization methods have already been proposed by researchers. Sahoo and Pal [26] used the Taguchi based Grey relational analysis to find out the optimum combination of parameters for better tribological performance of Ni-P coatings. Karaboga and Akay [27] used artificial bee colony (ABC) algorithm for optimizing a large number of numerical functions and compared the result obtained from ABC with that of genetic algorithm (GA), differential evolution (DE), and evolution strategy (ES) algorithms. They concluded that its performance is better than or similar to that of other population based algorithms although it uses less control parameters and it can be efficiently used for solving multiresponse optimization problems. In the present study, electroless Ni-P-Cu coating has been developed on mild steel substrate and artificial bee colony (ABC) algorithm is employed to find out the optimum combination of coating process parameters for better tribological properties (minimum friction and wear). The composition and surface morphology of the coating have been studied by energy dispersive X-ray analysis (EDX) and scanning electron microscopy (SEM).

## 2. Basic Considerations

### 2.1. Artificial Bee Colony (ABC) Algorithm

Inspired by the intelligent foraging behavior of honey bees, Karaboga and Basturk [28, 29] introduced the ABC algorithm for optimizing numerical problems. It can be noted that three parameters are of prime importance in the foraging behavior of honey bees, that is, food source (nectar), employed foragers, and unemployed foragers, and the foraging behavior leads to two modes, that is, recruitment of nectar source and abandonment of nectar source. In ABC, the colony of artificial bees contains generally two groups of bees: employed bees and onlooker bees. The employed bees have all the idea about the food source (nectar position) and quality of food (nectar amount). In the hive, all the employed bees with all their information of foods started waggle dance. This dance is the indication of amount as well as the quality of foods searched by the employed bees. On the other hand, in the hive there are also some unemployed bees, called onlooker bees. They watch the waggle dance and get the information about all the food sources and attracted to the best food source. In the next stage, the onlooker bees become employed and they started consuming the nectar from the best food source. When this food source becomes abandoned, the employed bee becomes a scout bee and starts to find new food source. As early as a scout finds a new food source, it becomes an employed bee and the cycle goes on until the best food source (optimum solution) is obtained. In the ABC algorithm, the number of employed bees and onlooker bees is equal to the number of solutions in the population. In the initialization step, the algorithm generates randomly distributed predefined number of initial food source (solution). Since each food source *X*_*i*_ is a solution vector to the optimization problem, each *X*_*i*_ vector holds *n* variables, (*X*_*ij*_, *j* = 1,…, *n*), which are to be optimized. After initialization, the solutions are subjected to repeated cycles *C* = 1,…, MCN (maximum cycle number). This is for the search process of the employed bees, onlooker bees, and scout bees. In the next step, that is, employed bees phase, the employed bees search for new food sources (*V*_*ij*_) having more nectar within the neighborhood of the food source (*X*_*ij*_) in their memory. They find a neighbor food source and then evaluate its profitability (fitness). The neighbor food source (*V*_*ij*_) is determined by using the formula given by

(1) $$\begin{array}{c} V_{ij}=X_{ij}+r_{ij}\left(X_{ij}-X_{kj}\right), \end{array}$$

where *X*_*kj*_ is the randomly selected food source, *i* is randomly chosen parameter index provided *k* ≠ *i*, and *r*_*ij*_ is a random number within the range of (0, 1). After producing the new food source (*V*_*ij*_), its fitness is calculated and a greedy selection is applied between *V*_*ij*_ and *X*_*ij*_. This fitness value is the indication of waggle dance of the employed bee. In the third step, the employed bees share their food source information with onlooker bees waiting in the hive and then onlooker bees choose a food source depending on the probability values calculated using the fitness values provided by employed bees. The probability value *P*_*i*_ with which *X*_*i*_ is chosen by an onlooker bee can be calculated by

(2) $$\begin{array}{c} P_{i}=\frac{\text{fitnes}{\text{s}}_{i}\left(x_{i}\right)}{\sum_{i=1}^{n}\text{fitnes}{\text{s}}_{i}\left(x_{i}\right)}. \end{array}$$

After a food source *X*_*i*_ for an onlooker bee is probabilistically chosen, a neighbourhood source *V*_*i*_ is determined by using (1), and its fitness value is computed. As in the employed bees phase, a greedy selection is applied between *V*_*i*_ and *X*_*i*_. Hence, more onlookers are recruited to richer sources and positive feedback behavior appears. Employed bees whose solutions cannot be improved through a predetermined number of trials, specified by the user of the ABC algorithm and called “limit” or “abandonment criteria” herein, become scouts and their solutions are abandoned. Then, the converted scouts start to randomly search for new solutions. For instance, if solution *X*_*m*_ has been abandoned, it is replaced by the new solution discovered by the scout. Then, the scout becomes employed bee. The artificial bee colony algorithm including main phases is given as follows.

*Algorithm*

(1) Initialize the population of solution *X*_*i*_.
(2) Evaluate the population, cycle = 1, and *k* = 0.
(3) Memorize the best solution, *X*_best_, and set *X*_best1_ = *X*_best_.
(4) Repeat (exploration phase).
(5) Produce new solutions *X*_new_ = *V*_*i*_ for the employee bees and evaluate them.
(6) Apply the greedy selection process for the employed bees.
(7) Rank the population and calculate the fitness.
(8) Calculate the probability *P*_*i*_ for the solution *X*_*i*_.
(9) Produce the new solutions *V*_*i*_ for the onlookers from the solution selected depending on *P*_*i*_ and evaluate them.
(10) Apply the greedy selection process for the onlookers.
(11) Determine the abandoned solution for the scout if existing, and replace it with a new randomly produced solution *X*_*i*_.
(12) Memorize the best solution *X*_best_ achieved so far.
(13) Set *k* = *k* + 1; cycle⁡ = cycle⁡+1.
(14) Until termination condition is met; i.e., cycle = MCN.

### 2.2. Choice of Design Parameters

Design of experiment (DOE) is an important part of any optimization problem. By applying appropriate design of experiment, time and cost can be saved. The first and foremost task in design of experiment is the selection of design parameters. In this optimization problem, after a large number of experimental trials, four most important design parameters have been selected. The first three parameters are coating parameters, namely, concentration of nickel sulphate, concentration of sodium hypophosphite solution, and concentration of copper sulphate solution; the fourth one is the postdeposition heat treatment temperature. Each of these four parameters is divided into three equally spaced levels within the operating range. The operating range is selected based on experimental trial, within which the coating can be deposited. The four main design parameters with their ranges and levels are shown in Table 1.

### 2.3. Taguchi Design of Experiment

This is an optimization problem with four design parameters and each has three levels, so with all possible combination of these parameters a total number of 81 (3^4^) experiments can be performed. In this experimental study, to save the time and cost, the Taguchi design of experiment is opted, as it is a robust design of experiment method. In Taguchi design, there are several numbers of standard orthogonal arrays (OA). The selection of orthogonal array depends on the total degrees of freedom of the experiment. The rule is that the degrees of freedom of the OA must be greater than or equal to the total degrees of the experiment. The degree of freedom of any factor with “*n*” level is (*n* − 1). In this present optimization problem, the degrees of freedom of all four individual factors are 8 [4 × (3 − 1)] and the three two-way interactions are 12 [3 × (3 − 1)×(3 − 1)]. Thus, the total degrees of freedom of the experiment are 20 (8 + 12). Hence, L_27_ OA is selected as it has 26 degrees of freedom. The L_27_ OA consists of 27 rows. Each row represents the combination of factors for deposition of coating and it has 13 columns representing the individual factors and their interactions.  Table 2 shows the standard L_27_ OA.

## 3. Experimental Details

### 3.1. Preparation of Substrate and Coating Deposition

**Figure 1:**
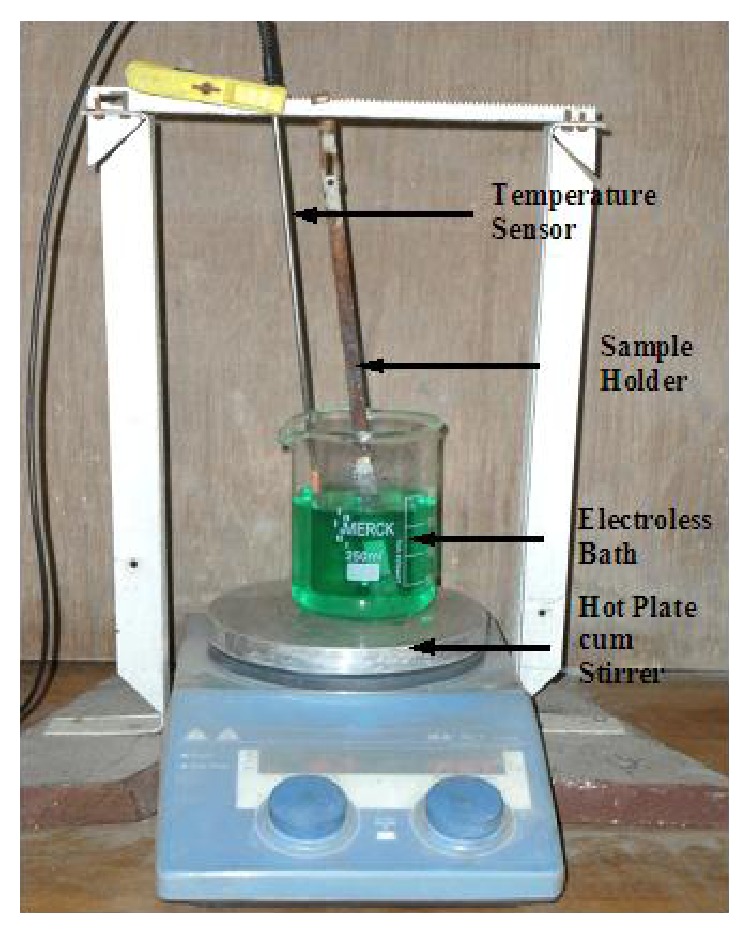
Experimental setup for electroless Ni-P-Cu coating deposition.

Mild steel blocks (AISI 1040) of size 20 mm × 20 mm × 8 mm are used as substrates for the deposition of electroless Ni-P-Cu coating. This particular dimension of the sample is chosen to fit the counter part of block on roller multitribotester apparatus. The sample is mechanically cleaned from foreign matters and corrosion products. After that, the MS sample is cleaned using distilled water. Then, a pickling treatment is given to the specimen with dilute (50%) Hydrochloric acid for one minute to remove any surface layer formed like rust followed by rinsing in distilled water and methanol cleaning.  Table 3 lists the bath composition and the operating conditions for successful coating of electroless Ni-P-Cu. Nickel sulphate is used as the source of nickel, while sodium hypophosphite is the reducing agent and sodium citrate was added as complexing agent. The bath is prepared by adding the constituents in appropriate sequence. The pH of the solution is maintained around 9.5 by continuous monitoring with a pH meter. The cleaned samples are activated in palladium chloride solution at a temperature of 55°C. Activated samples are then submerged into the electroless bath which is maintained at a temperature 85°C with the help of a hot plate cum stirrer attached with a temperature sensor also submerged in the solution. The deposition is carried out for a period of 2 hours. The range of coating thickness is found to lie around 28–30 microns by measuring with a digital micrometer. After deposition, the samples are taken out of the bath and heat treatment has been done in a box furnace according to the experimental design. After attaining the desired temperature, the samples are inserted into the furnace and kept for 1 hour. After that, the samples are allowed to cool slowly within the furnace. The experimental setup is shown in Figure 1. The average roughness of the coated specimen lies within the range of 0.8–1.28 *μ*m before wear and friction test.

### 3.2. Surface Morphology and Composition Study

**Figure 2:**
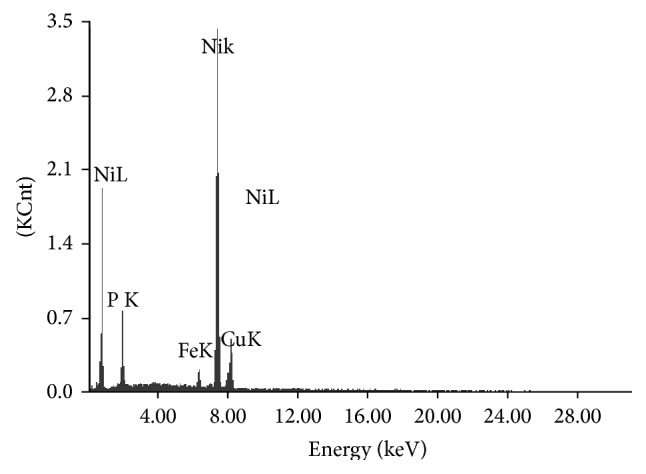
EDX spectra of Ni-P-Cu coated surface.

**Figure 3:**
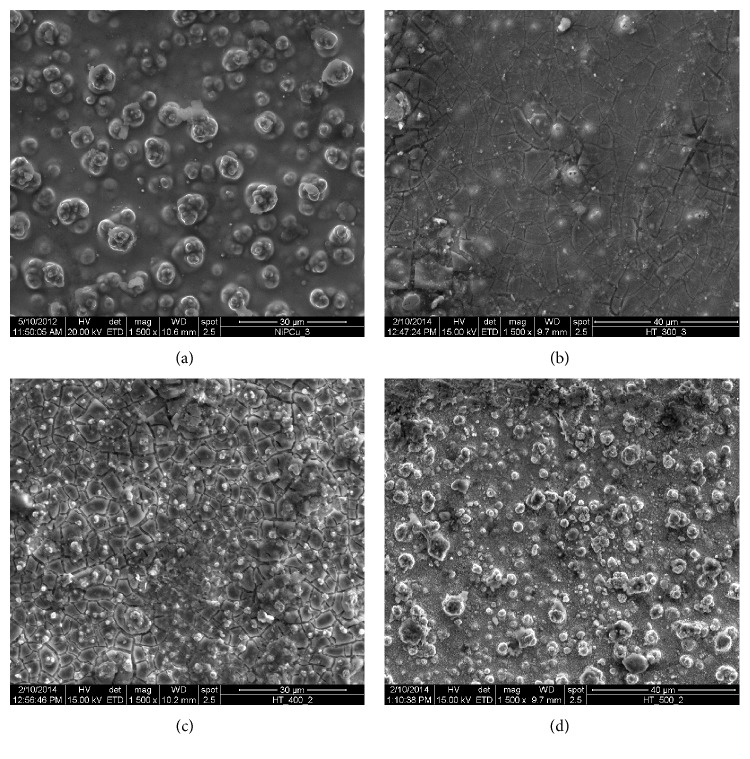
SEM image of Ni-P-Cu coated surface, (a) as deposited; (b) heat treated at 300°C; (c) heat treated at 400°C; (d) heat treated at 500°C.

**Figure 4:**
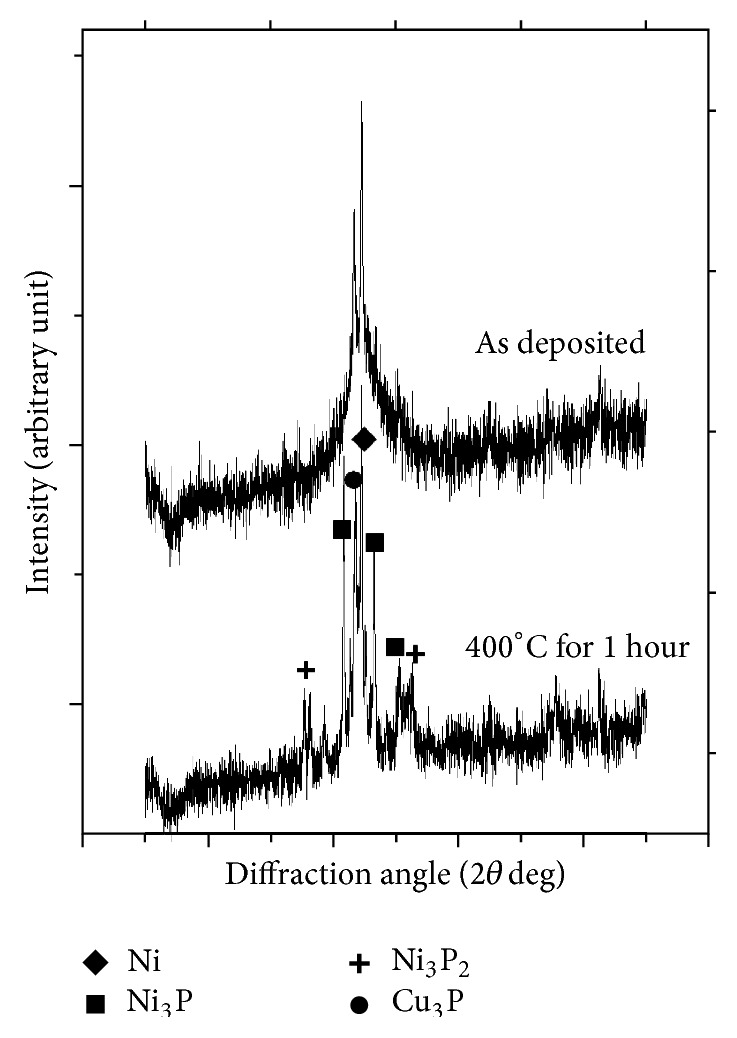
XRD spectrum of Ni-P-Cu coated surface.

The characterization of the coating is necessary so that it can be made sure that the coating is properly developed. Energy dispersive X-ray analysis (EDAX Corporation) is performed to determine the composition of the coating in terms of the weight percentages of nickel, phosphorous, and copper.  Figure 2 shows the EDX spectra of the coated surface. From the analysis, it is found that the coating consists of 11% P, 4% Cu, and the remaining is Ni.  Figure 3 shows the scanning electron micrograph (SEM) of as-deposited and heat treated (300°C, 400°C, 500°C) Ni-P-Cu coated surface. It is clear that the deposit has coarse nodular structure without any porosity in as-deposited condition. Nodular deposition in a coating depends on nucleation rate and the growth of the deposit. Nucleation rate depends on the bath constituents and the operating condition of the experiment. When it is heat treated at various temperatures, the globules change in size and transform into coarse grained structure leading to less harder deposit having low wear resistance. Form the figures it is clear that due to heating, crack appears in the coating. The phase transformation behavior has been studied by X-ray diffraction analysis (XRD).  Figure 4 shows the XRD pattern of as-deposited and heat treated condition. From the figure, it is clear that in as-deposited condition only very few broad crystalline peaks appear, so it may be said that it is a mixture of amorphous and crystalline structure in as-deposited condition. But, after heating, the broad crystalline peaks appear and the coating transforms into crystalline structure. The peaks are identified by computer algorithm, allowing rapid peak matching according to the joint committee on powder diffraction standards (JCPDS). The major crystalline peaks of Ni, Cu_3_P, Ni_3_P, and Ni_3_P_2_ appear after heating at 400°C for 1 hour.

### 3.3. Wear and Friction Measurement

Wear depths of heat-treated Ni-P-Cu coated specimens are measured using a multitribotester with block on roller configuration (DUCOM, TR-25) under nonlubricated condition. The Ni-P-Cu coated specimens serve as test specimens which are held horizontally against a rotating roller of 50 mm diameter × 20 mm thickness. The wear test of each specimen is carried out for 5 minutes with 25 N load at a speed of 50 r/min. The steel roller is coated with titanium nitride of hardness 85 HRc, which is higher than the hardness of the Ni-P-Cu coated specimen (42 HRc) in order to ensure that wear will take place only in the test specimens. Dead weights are placed on the loading platform, which is attached to one end of a 1 : 5 ratio loading lever. Wear is measured in terms of displacement with the help of linear voltage resistance transducer. It is worth noting that, in general, wear is measured in terms of wear volume or mass loss. But, in the present case, wear is expressed in terms of displacement or wear depth. Hence, to ensure that the wear measurements are accurate, the displacement results for wear are compared with the weight loss of the specimens and almost linear relationship is observed between the two for the range of test parameters considered in the present study. The frictional force is measured by a frictional force sensor that uses a beam type load cell of 1000 N capacity. The speed of the roller and the duration of tests can be controlled via a computer attached to the tribotester.

## 4. Results and Discussion

### 4.1. Analysis of Responses

This is an optimization problem dealing with four input parameters, namely, concentration of nickel sulphate solution (*x*_1_), concentration of sodium hypophosphite solution (*x*_2_), concentration of copper sulphate solution (*x*_3_), and postdeposition heat treatment temperature (*x*_4_), and two output parameters, namely, wear depth (*W*) and coefficient of friction (COF). The results of friction and wear test of all the 27 experiments are shown in Table 4. The wear depth and coefficient of friction of each of 27 electroless Ni-P-Cu coated specimens are measured and the relation between the input design parameters and the output parameters, namely, wear depth (*W*) and coefficient of friction (COF), has been developed separately by polynomial regression equations using Minitab software. The equations are follows:

(3) W=25.7209−0.444082x1−2.54733x2 −68.6768x3+0.162786x4−0.0530553x12 +0.0343005x1x2+1.73643x1x3+0.00554055x1x4 +0.144575x22−4.34993x2x3−0.00119613x2x4 +57.1921x32+0.0528007x3x4−0.000425935x42,

(4) COF=2.04438+0.0278959x1−0.0658979x2 −1.16193x3−0.00686096x4−0.000776911x12 +0.000180347x1x2+0.029806x1x3 +0.0000184573x1x4+0.000718156x22 −0.00774467x2x3+0.000118425x2x4 −0.0728194x32+0.0011301x3x4+0.0000048783x42.

The coefficients of determination (*R* squared) values are 88.32% for (3) and 83.27 for (4), and the adjusted *R* squared values are 84.17% and 80.03% for those equations, respectively. As this is a multiobjective optimization problem, instead of taking the responses separately, both the responses have been optimized together. Here, the objective function taking both the responses simultaneously has been developed according to past research [30]. The multiresponse objective function is given by

(5) $$\begin{array}{c} f=\frac{w_{1}\cdot W}{W_{\min}}+\frac{w_{2}\cdot \text{COF}}{\text{CO}{\text{F}}_{\min}}, \end{array}$$

where *W*_min⁡_ and COF_min⁡_ are the minimum values of wear and coefficient of friction, respectively. From Table 4, *W*_min⁡_ = 1.432 *μ*m and COF_min⁡_ = 0.103. *w*_1_ and *w*_2_ are the weights assigned to wear and coefficient of friction, respectively. The weight values are assigned in such a way that they always satisfy the condition *w*_1_ + *w*_2_ = 1. The values of these weights are dependent on the priority of the response. In the present study, both the responses are given the same priority; hence, the same weights are selected for both; that is, *w*_1_ = *w*_2_ = 0.5. “*W*” and “COF” are the polynomial functions mentioned above. As both the responses, that is, wear depth and coefficient of friction, are to be minimized, hence the multiresponse function “*f*” is to be minimized. This objective function has been solved by artificial bee colony algorithm with the following parameters:

 number of population/swarm size = 6,
 number of employed bees = 50% of population,
 number of onlooker bees = 50% of population,
 number of scouts per cycle = 1,
 number of cycles = 1000,
 limit = 500.

**Figure 5:**
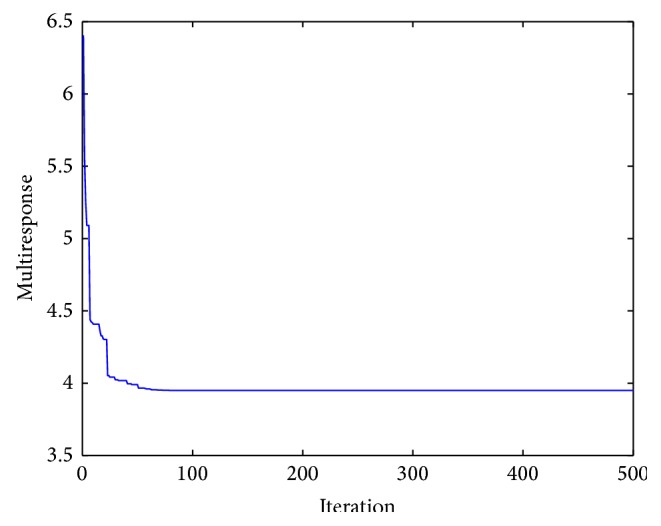
Variation of multiresponse with iterations.

**Figure 6:**
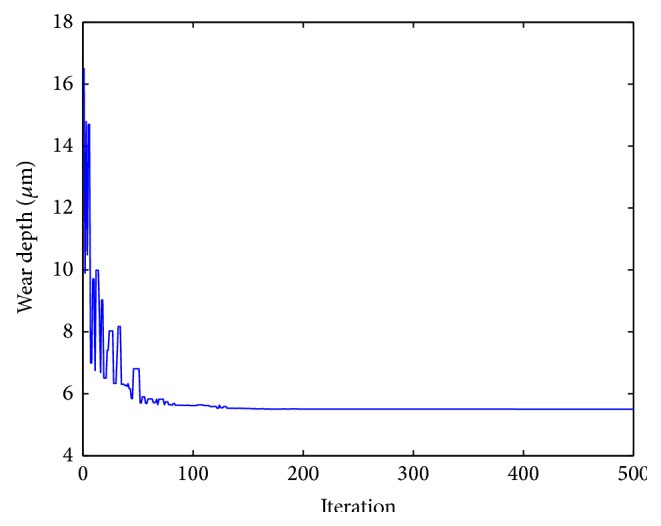
Variation of wear depth with iterations.

**Figure 7:**
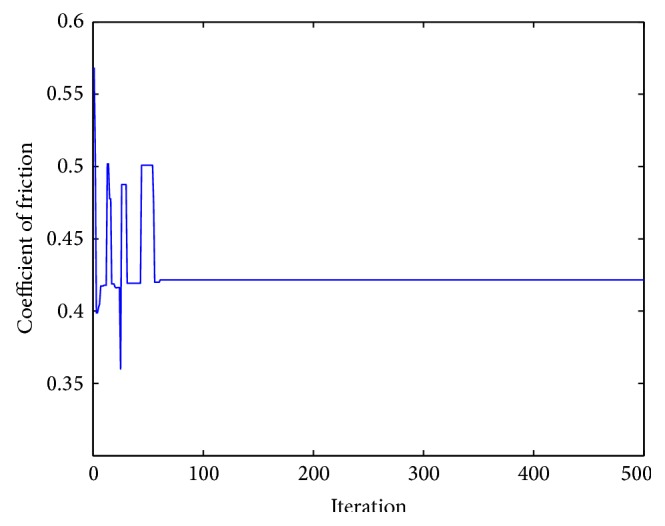
Variation of COF with iterations.

**Figure 8:**
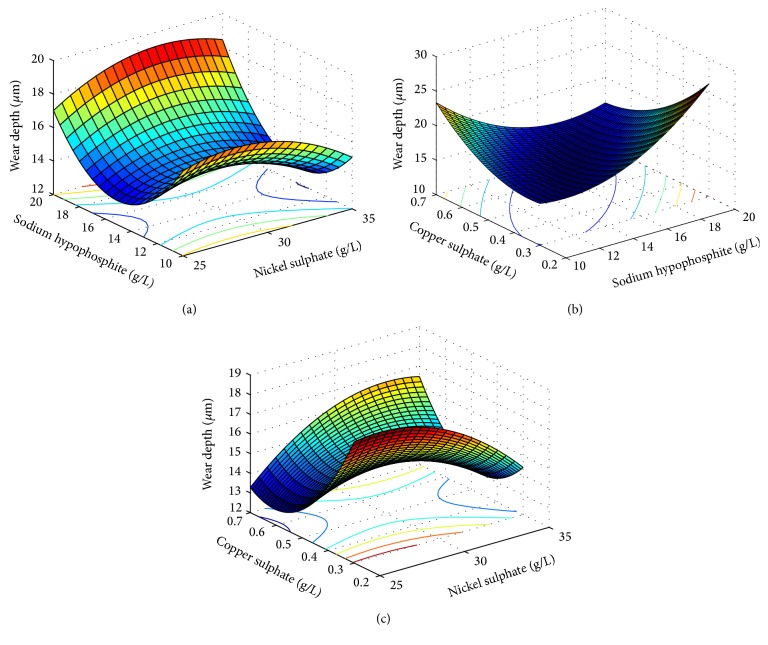
Variation of wear depth with coating parameters.

**Figure 9:**
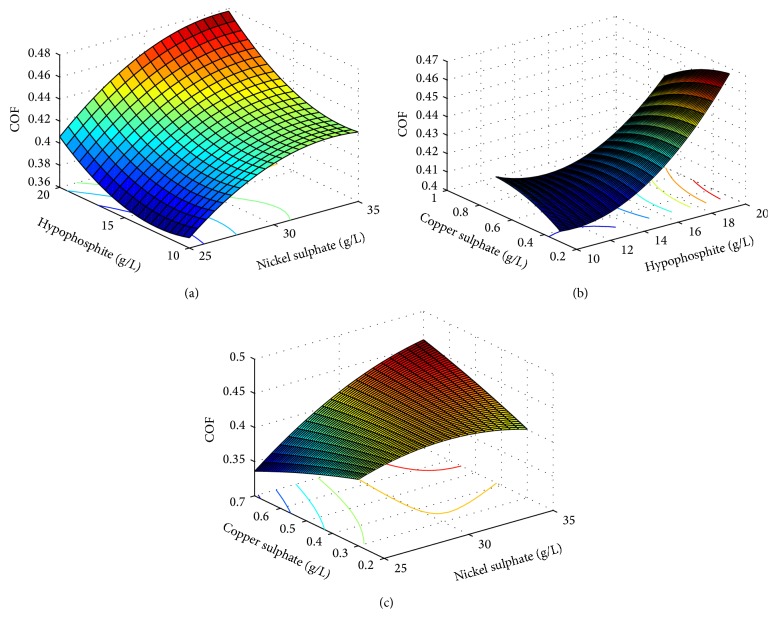
Variation of COF with coating parameters.

From the analysis, the optimum values of the input parameters are obtained. The values are concentration of nickel sulphate 25.0000 g/L, concentration of hypophosphite 16.6165 g/L, concentration of copper sulphate 0.6289 g/L, and heat treatment temperature 500°C. The corresponding values of the output parameters are wear depth 5.40 *μ*m, coefficient of friction 0.42, and the multiresponse function *f* = 3.93.  Figure 5 shows the convergence of the multiresponse value with the number of iterations. Figures 6 and 7 show the variation of wear and coefficient of friction with the number of iterations. From these figures, it is clear that the variation of wear depth and coefficient of friction is not very uniform. It is because a certain combination of input parameters may give a low value of wear depth but the same combination may yield a high value of coefficient of friction. As this is a multiresponse optimization problem, the aim is to find the best possible combination of input parameters for which the wear depth and coefficient of friction will have optimum values and the multiresponse function has a minimum value. Figures 8 and 9 show the variation of wear depth and COF with the variation of coating parameters, respectively.

**Figure 10:**
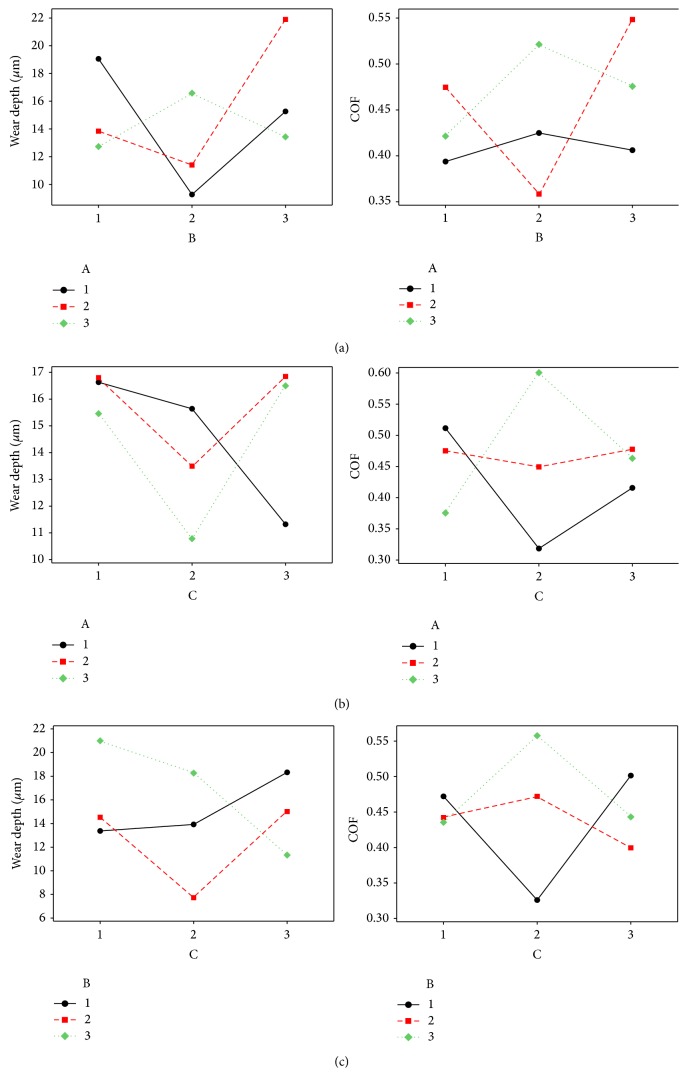
Interaction plots of parameters, (a) A versus B, (b) A versus C, and (c) B versus C.

The interaction plots of the three coating parameters reveal the relative significance of interaction of parameters. If the lines of the interaction plots are nonparallel to each other, then there exist strong interactions among the parameters.  Figure 10 shows the interaction plots for both the responses. From the interaction plots, it is clear that, for wear, interaction between nickel and hypophosphite plays a vital role, whereas, for COF, interaction between nickel-copper and hypophosphite-copper has significant effect.

### 4.2. Tribological Behavior

**Figure 11:**
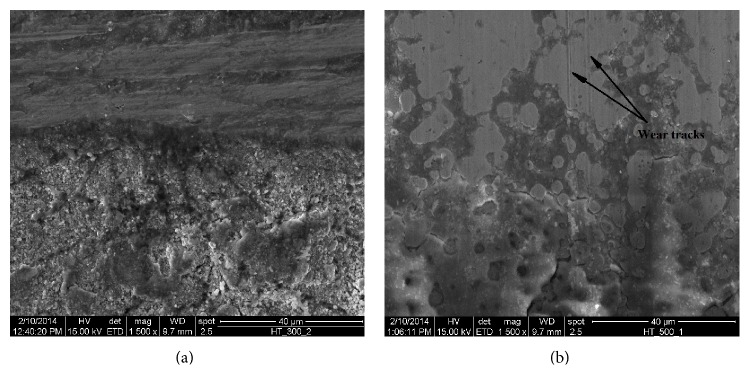
SEM image of Ni-P-Cu coated surface after wear test.

Figure 11 shows SEM image of worn surface at different magnification. It is evident from Figure 11(a) that, at the contact area of roller and the specimen, a bulk amount of material has come out by shearing in the form of wear debris and some loose wear particles are formed at the worn region, which leads to adhesive wear being predominant in this case. In some other part of the worn surface shown in Figure 11(b), wear tracks are clearly visible as a result of ploughing effect and plastic deformation of asperities present in the coated surface. As the surface of the rotating roller is hard and smooth, the ploughing effect may form due to the entrapped hard particles between the sliding surfaces. This phenomenon means that three-body abrasion is predominant in this case. Hence, the wear behavior of heat treated electroless Ni-P-Cu surface includes both the adhesive and abrasive wear mechanisms.

### 4.3. Confirmation Test

A confirmation test has been carried out to check whether the result obtained from the analysis is practical or not. With the combination of optimum input parameters obtained from the analysis, a coating has been deposited and the tribological test carried out with the same parameters. The exact values of these parameters were determined with the help of a digital balance, able to measure up to five decimal places. The result obtained from the confirmation experiment shows good agreement with that of analytical result. The result of confirmation experiment is shown in Table 5. From the table, it is clear that the experimental result varies 6% in wear depth and 7% in COF from the analytical result.

## 5. Conclusions

In this paper, electroless ternary Ni-P-Cu coating has been developed on mild steel substrate by varying four design parameters, namely, concentration of nickel source (nickel sulphate), concentration of reducing agent (sodium hypophosphite), concentration of copper source (copper sulphate), and postdeposition heat treatment temperature. The wear depth and coefficient of friction of the coated surface are measured with a multitribotester instrument with block on roller configuration. The result obtained shows that a huge variation of wear is obtained with the variation of nickel sulphate and copper sulphate concentration, but the coefficient of friction varies largely with the variation of nickel sulphate and sodium hypophosphite concentration. Artificial bee colony algorithm is successfully employed for finding out the optimal combinations of the design process parameters of electroless Ni-P-Cu coatings for minimum wear depth and coefficient of friction simultaneously. From the analysis, the optimum combination is obtained as 25.0000 g/L nickel sulphate, 16.6165 g/L sodium hypophosphite, 0.6285 g/L copper sulphate, and 500°C heat treatment temperature; that is, lower value of nickel sulphate, medium value of sodium hypophosphite, higher value of copper sulphate, and higher value of heat treatment temperature give a better tribological result. The optimum value obtained from the analysis shows a good agreement with that of experimental value. The energy dispersive X-ray analysis shows that it is a pure ternary coating consisting of nickel phosphorous and copper. The surface morphology is studied by SEM which reveals that wear behavior of heat treated electroless Ni-P-Cu surface includes both the adhesive and abrasive wear mechanisms. XRD analysis reveals that in as-deposited condition the coating is a mixture of amorphous and crystalline structure and after heat treatment it transforms into crystalline structure.

## Figures and Tables

**Table 1:** Main coating parameters with their levels.

Design Factors	Unit	Levels		
		1	2	3
Concentration of source of nickel (nickel sulphate solution)	g/L	25	30	35
Concentration of reducing agent (sodium hypophosphite solution)	g/L	10	15	20
Concentration of source of copper (copper sulphate)	g/L	0.3	0.5	0.7
Heat treatment temperature	°C	300	400	500

**Table 2:** L_27_ orthogonal array.

Trial Number	Column numbers												
	1 (A)	2 (B)	3 (A × B)	4 (A × B)	5 (C)	6 (A × C)	7 (A × C)	8 (B × C)	9 (D)	10	11 (B × C)	12	13
1	1	1	1	1	1	1	1	1	1	1	1	1	1
2	1	1	1	1	2	2	2	2	2	2	2	2	2
3	1	1	1	1	3	3	3	3	3	3	3	3	3
4	1	2	2	2	1	1	1	2	2	2	3	3	3
5	1	2	2	2	2	2	2	3	3	3	1	1	1
6	1	2	2	2	3	3	3	1	1	1	2	2	2
7	1	3	3	3	1	1	1	3	3	3	2	2	2
8	1	3	3	3	2	2	2	1	1	1	3	3	3
9	1	3	3	3	3	3	3	2	2	2	1	1	1
10	2	1	2	3	1	2	3	1	2	3	1	2	3
11	2	1	2	3	2	3	1	2	3	1	2	3	1
12	2	1	2	3	3	1	2	3	1	2	3	1	2
13	2	2	3	1	1	2	3	2	3	1	3	1	2
14	2	2	3	1	2	3	1	3	1	2	1	2	3
15	2	2	3	1	3	1	2	1	2	3	2	3	1
16	2	3	1	2	1	2	3	3	1	2	2	3	1
17	2	3	1	2	2	3	1	1	2	3	3	1	2
18	2	3	1	2	3	1	2	2	3	1	1	2	3
19	3	1	3	2	1	3	2	1	3	2	1	3	2
20	3	1	3	2	2	1	3	2	1	3	2	1	3
21	3	1	3	2	3	2	1	3	2	1	3	2	1
22	3	2	1	3	1	3	2	2	1	3	3	2	1
23	3	2	1	3	2	1	3	3	2	1	1	3	2
24	3	2	1	3	3	2	1	1	3	2	2	1	3
25	3	3	2	1	1	3	2	3	2	1	2	1	3
26	3	3	2	1	2	1	3	1	3	2	3	2	1
27	3	3	2	1	3	2	1	2	1	3	1	3	2

**Table 3:** Electroless bath constituents.

Parameters	Values
Nickel sulphate (g/L)	25–35
Sodium hypophosphite (g/L)	10–20
Sodium citrate (g/L)	15
Copper sulphate (g/L)	0.3–0.7
pH	9.5
Temperature	85°C
Duration of coating	2 hrs
Bath volume (mL)	200

**Table 4:** Result of friction and wear test.

Serial Number	Wear depth (*μ*m)	COF
1	18.980	0.571
2	26.617	0.103
3	11.564	0.520
4	10.464	0.459
5	4.322	0.368
6	13.035	0.457
7	20.448	0.487
8	15.979	0.482
9	9.369	0.261
10	9.762	0.539
11	13.653	0.340
12	18.109	0.551
13	14.699	0.459
14	1.432	0.374
15	18.086	0.259
16	25.949	0.413
17	25.396	0.623
18	14.349	0.609
19	11.369	0.306
20	1.497	0.536
21	25.320	0.433
22	18.414	0.409
23	17.428	0.674
24	13.909	0.483
25	16.590	0.406
26	13.427	0.567
27	10.262	0.460

**Table 5:** Result of confirmation test.

Input parameters	Value	Result from ABC analysis		Result from confirmation test	
		Wear depth (*μ*m)	COF	Wear depth (*μ*m)	COF
Concentration of nickel sulphate solution (g/L)	25.0000	5.40	0.42	5.72	0.45
Concentration of sodium hypophosphite solution (g/L)	16.6165				
Concentration of copper sulphate (g/L)	0.6289				
Heat treatment temperature (°C)	500				
